# Echocardiographic assessment of pulmonary vascular resistance in pulmonary arterial hypertension

**DOI:** 10.1186/1476-7120-8-21

**Published:** 2010-06-07

**Authors:** Vincent Roule, Fabien Labombarda, Arnaud Pellissier, Rémi Sabatier, Thérèse Lognoné, Sophie Gomes, Emmanuel Bergot, Paul Milliez, Gilles Grollier, Eric Saloux

**Affiliations:** 1 Department of Cardiology, University Hospital of Caen, 14033 Caen Cedex, France; 2 Department of Pneumology, University Hospital of Caen, 14033 Caen Cedex, France

## Abstract

**Background:**

Echocardiographic ratio of peak tricuspid regurgitant velocity to the right ventricular outflow tract time-velocity integral (TRV/TVI rvot) was presented as a reliable non-invasive method of estimating pulmonary vascular resistance (PVR). Studies using this technique in patients with moderate to high PVR are scarce. Left ventricular outflow tract time-velocity integral (TVI lvot) can be easier to measure than TVI rvot, especially in patients with severe pulmonary hypertension (PH) with significant anatomical modifications of the right structures.

**Aims:**

We wanted to determine whether the TRV/TVI rvot and TRV/TVI lvot ratios would form a reliable non-invasive tool to estimate PVR in a cohort of patients with moderate to severe pulmonary vascular disease.

**Methods:**

Doppler echocardiographic examination and right heart catheterisation were performed in 37 patients. Invasive PVR was compared with TRV/TVI rvot and TRV/TVI lvot ratios using regression analysis. Two equations were modelled and the results compared with invasive measurements using the Bland-Altman analysis. Using receiver-operating characteristics curve analysis, a cut-off value for the two ratios was generated.

**Results:**

Correlation coefficients between invasive PVR and TRV/TVI rvot then TRV/TVI lvot were respectively 0.76 and 0.74. Two new equations were found but the Bland-Altman analysis showed wide standard deviations (respectively 3.8 and 3.9 Wood units). A TRV/TVI rvot then TRV/TVI lvot ratio cut-off value of 0.14 had a sensitivity of 93% and a specificity of 57% for the first and a sensitivity of 87% and a specificity of 57% for the second to determine PVR > 2 Wood units.

**Conclusion:**

Echocardiography is useful for the screening of patients with pulmonary hypertension and PVR > 2 WU. It remains disappointing for accurate assessment of high PVR. TVI lvot may be an alternative to TVI rvot for patients for whom accurate TVI rvot measurement is not possible.

## Introduction

Assessment of pulmonary vascular resistance (PVR) is crucial in the diagnosis and management of cardio pulmonary diseases such as pulmonary arterial hypertension (PH). Invasive measurement of PVR by right heart catheterisation remains the gold standard method [1,2]. Echocardiographic estimation of PVR using the ratio of peak tricuspid regurgitant velocity (TRV) to the right ventricular outflow tract time-velocity integral (TVI rvot) was presented as a reliable non-invasive method to determine PVR [3]. While the ability of Doppler measurement appeared criticable in patients with high PVR [4], few studies have evaluated the TVR/TVI rvot ratio in these cohorts of patients. Left ventricular outflow tract time-velocity integral (TVI lvot) can be easier to measure than TVI rvot, especially in patients with severe PH with important anatomical modifications of the right structures which may make accurate measurements of the TVI rvot difficult. Nevertheless TRV/TVI lvot was never tested as an alternative to TRV/TVI rvot.

We aimed to determine whether the TRV/TVI rvot ratio would form a reliable non-invasive tool to estimate PVR in a cohort of patients with moderate to severe pulmonary vascular disease and whether TRV/TVI lvot ratio could be an alternative.

## Methods

### Study population

Patients monitored in our Pulmonary Hypertension centre were prospectively recruited. All patients underwent right-heart catheterisation within 24 hours (mean time: 6 hours) of the time of their echocardiography. Exclusion criteria included tricuspid regurgitation grade > 2, intra-cardiac shunt, aortic stenosis or aortic insufficiency grade > 2 to avoid modifying the TVI rvot or TVI lvot measurement. All subjects gave their written informed consent for the participation to the study.

### Cardiac catheterisation

Patients underwent standard right heart catheterisation using a Swan-Ganz catheter with jugular or femoral venous access. The following pressure measurements were obtained: right atrial pressure (RAP), right ventricular pressure (RVP), pulmonary artery systolic pressure (PASP), pulmonary artery diastolic pressure (PADP), mean pulmonary artery pressure (MPAP) and right pulmonary capillary wedge pressure (PCWP). Cardiac output (Qp) was determined by thermodilution (mean of three consecutive measurements without variation > 10%). The PVR was calculated using the formula: PVR = (MPAP - PCWP)/Qp.

### Echocardiography

Echocardiographic assessment was performed using a Sonos 5500 ultrasound system (Philips^®^) equipped with an S3 transducer, before catheterisation. The TVI rvot (cm) was obtained by placing a pulsed wave Doppler sample volume just within the pulmonary valve from the parasternal short axis view. TRV (m/s) was obtained using continuous wave Doppler from apical 4-chamber, parasternal and subcostal views and the highest peak value was retained. TVI lvot was obtained from the apical 5-chamber view by placing a pulsed wave Doppler sample volume in the left ventricular outflow tract just below the aortic valve. TVI rvot and TVI lvot were measured 3 times and averaged. Left ventricular ejection fraction (LVEF) was assessed from an apical 4-chamber view using the monoplane modified Simpson's rule. All measurements were made off-line. The sonographer was blinded to the results of cardiac catheterization. Contrast echocardiography (e.g. agitated saline) was not used.

### Statistical analysis

All statistical analyses were performed using StatView version 5.0. Continuous variables were expressed as mean ± SD. Linear regression analysis was generated between invasive PVR and TRV/TVI rvot and between invasive PVR and TRV/TVI lvot, leading to Pearson's correlation coefficient. Two equations derived from TRV/TVI rvot and TRV/TVI lvot were obtained. The relation between calculation of PVR by catheter measurements and Doppler echocardiography with the two methods was assessed by Bland-Altman analysis. Receiver operating characteristic curves were generated to determine a cut-off value for TRV/TVI rvot and for TRV/TVI lvot with the best sensitivity to predict elevated PVR values (PVR > 2 WU). Linear regression analysis was realized between invasive cardiac output and TVI rvot then TVI lvot, and between invasive PASP and TRV, leading to Pearson's correlation coefficient. 40% of the Doppler images were re-evaluated to quantify the intra- and inter-observer reliability by calculating the intraclass correlation coefficient.

## Results

Overall, 37 consecutive patients (mean age 62 ± 12 years, 54% women) , diagnosed with pulmonary hypertension were enrolled in this prospective cohort between June 2005 and December 2007. Mean PVR was 4.8 ± 3 Wood units with preserved LVEF (mean LVEF: 66 ± 6%) and cardiac output. Invasive haemodynamic data are shown in Table 1. Referral diagnoses were idiopathic pulmonary hypertension (n = 11), portopulmonary hypertension (n = 7), chronic thromboembolic pulmonary hypertension (n = 6), pulmonary fibrosis (n = 5), systemic sclerosis (n = 5) and chronic obstructive pulmonary disease (n = 3). Clinical and echocardiographic data are resumed in Table 2. Most patients presented dilated right atrium and ventricle. Systolic velocity of the tricuspid annulus with Doppler tissue imaging was lower to 11.5 cm/s in 16 patients.

**Table 1:** Hemodynamic characteristics of the patients

Measurements	Results
PASP (mmHg)	71 ± 24.7
PADP (mmHg)	24.3 ± 8.9
MPAP (mmHg)	37.5 ± 12.8
PCWP (mmHg)	11.6 ± 2.8
RAP (mmHg)	9.6 ± 4.8
PVR (WU)	4.8 ± 3
Cardiac Output (L/min)	5.7 ± 2

COPD: Chronic Obstructive Pulmonary Disease; MPAP: Mean Pulmonary Artery Pressure; PADP: Pulmonary Artery Diastolic Pressure; PASP: Pulmonary Artery Systolic Pressure; PCWP: Pulmonary Capillary Wedge Pressure; PVR: Pulmonary Vascular Resistances; WU: Wood units; RAP: Right Atrial Pressure. Results were expressed as mean ± SD.

**Table 2:** Clinical and echocardiographic characteristics of the patients

Characteristics	Results
WHO functional class:	
- I	n = 2
- II	n = 16
- III	n = 15
- IV	n = 4
Rest rhythm:	
- atrial fibrillation	n = 5
- sinusal rhythm	n = 32
RAP (mmHg)	8.3 ± 3.9
PASP (mmHg)	64 ± 19.5
Right atrial area (cm2)	21 ± 6
Right ventricular end-diastolic diameter (cm)	3.8 ± 0.6
Tricuspid annular plane systolic excursion (cm)	2.1 ± 0.5
Systolic velocity of the tricuspid annulus (cm/s)	12.4 ± 2.8

PASP: Pulmonary Artery Systolic Pressure; RAP: Right Atrial Pressure.

**Figure 1:**
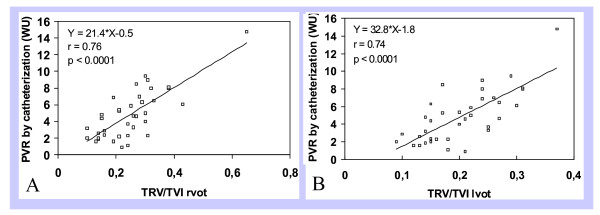
**Linear regression analysis showing correlation between PVR by catheterization in Wood units (WU) and ratios TRV/TVI rvot (Panel A) and TRV/TVI lvot (Panel B) obtained by echocardiography**. PVR = Pulmonary vascular resistance; TRV/TVI lvot = Peak tricuspid regurgitant velocity/Left ventricular outflow tract time-velocity integral; TRV/TVI rvot = Peak tricuspid regurgitant velocity/Right ventricular outflow tract time-velocity integral; WU = Wood units.

Linear regression analysis between invasive PVR and TRV/TVI rvot demonstrated good correlation for all patients (r = 0.76; p < 0.0001). Similarly, good correlation was found between invasive PVR and TRV/TVI lvot (r = 0.74; p < 0.0001) for all patients (Figure 1, Panels A and B).

The equations derived from these linear regressions were:(a)(b)

**Figure 2:**
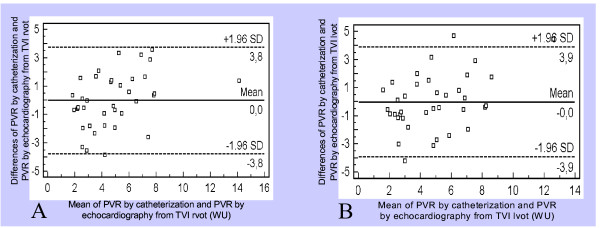
**Bland Altman analysis showing the limits of agreement between PVR obtained by catheterization and PVR obtained by echocardiography with TVI rvot (Panel A) and with TVI lvot (Panel B)**. PVR = Pulmonary vascular resistance; TRV/TVI lvot = Peak tricuspid regurgitant velocity/Left ventricular outflow tract time-velocity integral; TRV/TVI rvot = Peak tricuspid regurgitant velocity/Right ventricular outflow tract time-velocity integral; WU = Wood units.

**Figure 3:**
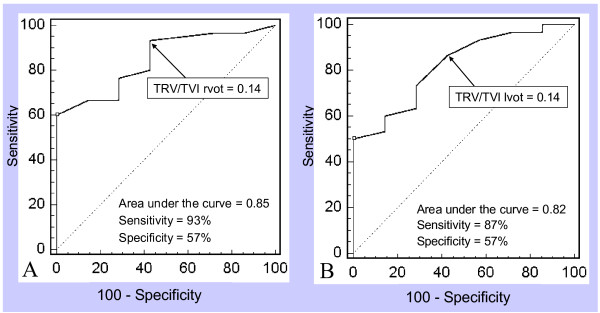
**Receiver operating characteristics (ROC) curves showing a sensitivity of 93% with a specificity of 57% for a TRV/TVI rvot cutoff value of 0.14 (Panel A, area under the curve = 0.85; p = 0.005) and a sensitivity of 87% with a specificity of 57% for a TRV/TVI lvot cutoff value of 0.14 (Panel B; area under the curve = 0.82; p = 0.009) to determine PVR > 2 WU**. PVR = Pulmonary vascular resistance; TRV/TVI lvot = Peak tricuspid regurgitant velocity/Left ventricular outflow tract time-velocity integral; TRV/TVI rvot = Peak tricuspid regurgitant velocity/Right ventricular outflow tract time-velocity integral; WU = Wood units.

Bland Altman analysis showed a homogeneous distribution with a difference of ± 3.8 Wood units for the equation using VTI rvot and ± 3.9 Wood units for that using VTI lvot (Figures 2, Panels A and B). A TRV/TVI rvot cut-off value of 0.14 provided a sensitivity of 93% with a specificity of 57% to determine PVR > 2 WU (area under the curve = 0.85). The same cut-off value of 0.14 for the ratio TRV/TVI lvot provided a sensitivity of 87% with a specificity of 57% (area under the curve = 0.82) (Figure 3, Panels A and B).

TRV in echocardiography and invasive PASP had a good correlation (R = 0.8). Correlation between invasive cardiac output and TVI rvot or TVI lvot was poor (respectively R = 0.37 and R = 0.44).

The intraclass coefficient correlation for the intra- and inter-observer reliabilities were 0.99 for both.

## Discussion

We found that TRV/TVI rvot and TRV/TVI lvot determined by transthoracic echocardiography were significantly correlated with invasive PVR in a cohort of patients with moderate and high PVR. However, the agreement between PVR determined by echocardiography and invasive PVR was poor.

Echocardiography plays a key role in the diagnosis, prognosis and follow-up of patients with PH. PASP measurement should ideally be completed by a PVR estimation [5]. First proposal of Doppler assessment of PVR was very interesting, showing excellent correlation with invasive measurements in patients with near normal or moderate level of right pressure and PVR [3]. Of the echocardiographic methods used to assess PVR, equations using the ratio TRV/TVI rvot remain the simplest and the most widely used. Nevertheless, studied in patients with high pulmonary pressure and PVR levels, the accuracy of this Doppler ratio in a practical daily activity was poor. In agreement with our results, TRV/TVI rvot was reported to be correlated significantly with invasively-determined PVR in populations with a similar level of PVR [4,6]. Using a cut-off value of 0.14 for these two ratios, patients with PVR greater than 2 Wood units could be identified with high sensitivity but poor specificity. This cut-off value remained close to previous values reported by several studies using TRV/TVI rvot [3,4,7]. While the TRV/TVI rvot may help to identify patients who have high PVR, we failed to calculate the PVR precisely. The inaccuracy of echocardiography to determe PVR was nevertheless emphasized, especially in patients with severe PH. This may be explained by the accumulation of potential sources of error in the estimation of PVR by echocardiography in these patients. In case of severe PH, in fact, small variations of TRV cause large changes in PASP value (since the modified Bernoulli equation is applied to TRV); and precise estimation of right atrial pressure may be more difficult when it is high. Rajagolopan *et al*. used this method to demonstrate that there was no linear or logarithmic relationship between TRV/TVI rvot and PVR in patients with PVR *> *8 Wood units [4]. Only Abbas *et al*. were able to report good agreement between invasive PVR and PVR evaluated by echocardiography in a cohort of patients less severe than ours with a mean PVR of 2 Wood units [3]. The threshold of 2 WU to determine pulmonary arterial hypertension is questionable and varies between studies [3-5,7-9]. Considering the weak specificity of PVR measurements by echocardiography and the poor prognosis of patients with PH, we think that it is important to choose the lower limit in order to minimise the number of false negatives.

Our results did not demonstrate any advantage derived by the use of both TVI rvot and TVI lvot. We estimate the best practical approach remains the use of TVI rvot in first intention as TRV/TVI rvot has more physiological basis than TRV/TVI lvot. Correct alignment of the ultrasound beam is a vital factor to ensure adequate determination of TVI rvot. Sometimes anatomical variations of the right-heart structures may make difficult accurate measurements of the TVI rvot in patients with PH, with a risk to introduce significant errors in right ventricular stroke volume estimation. Using TVI lvot to estimate right ventricular stroke volume may give more acurate estimation and may be an alternative, provided that no significant cardiac shunt or aortic valvulopathy are present.

While TRV in echocardiography and invasive PASP had a good correlation, correlation between invasive cardiac output and Doppler measurement (ie TVI rvot , TVI lvot) remained weak. Limits of our equations seems to be the assimilation of TVI to the cardiac output and not localization of its measurement. Contrary to a recent work which considered heart rate to estimate non invasively PVR [10], we did not take account the heart rate in equation. This may explain the low detected correlation between the cardiac output and the TVI lvot or TVI rvot.

Right heart catheterisation is indicated in all patients with suspected PH to confirm the diagnosis, assess the severity and test the vasoreactivity of the pulmonary circulation [2] but screening of these patients was previously done by echocardiography. PASP and tricuspid regurgitation velocity are recommanded for screening but could be inaccurate in some patients [2,5]. Estimation of echocardiographic PVR with ours equations increases sensibility. TVI rvot have to be used first being more validated in the literature [3-7,10,11] but TVI lvot remains an alternative.

### Limits

Echocardiography and right heart catheterization were not performed simultaneously in this study. We tried to minimize this potential source of error by performing the echocardiography before the invasive measurements under comparable loading conditions. Measurements of cardiac output using thermodilution may be problematic due to tricuspid regurgitation.We are aware of a quite significant fraction of patient with severe pulmonary hypertension were excluded of our study because of tricuspid regurgitation grade > 2. As severe tricuspid regurgitation is a well known pitfall of thermodilution cardiac output measurement, we prefered exclude these patients in order to obtain the most reliable invasive cardiac output.

## Conclusion

While TRV/TVI rvot and TRV/TVI lvot determined by transthoracic echocardiography significantly correlates with invasive PVR, accurate measurement of PVR by echocardiography remains disappointing, especially in case of a high level of PH. However, echocardiography can identify patients with PVR greater than 2 Wood units with high sensitivity and remains a clinically useful tool when screening patients with PH and high PVR.

## Abbreviations

COPD: Chronic obstructive pulmonary disease; LVEF: Left ventricular ejection fraction; MPAP: Mean pulmonary artery pressure; PH: Pulmonary arterial hypertension; PADP: Pulmonary artery diastolic pressure; PASP: Pulmonary artery systolic pressure; PCWP: Pulmonary capillary wedge pressure; PVR: Pulmonary vascular resistance; Qp: Cardiac output; RAP: Right atrial pressure; TRV: Peak tricuspid regurgitant velocity; TVI lvot: Left ventricular outflow tract time-velocity integral; TVI rvot: Right ventricular outflow tract time-velocity integral; WU: Wood unit

## Competing interests

The authors declare that they have no competing interest.

## Authors' contributions

VR participated in the echocardiographic measurements and drafted the manuscript. FL participated in the echocardiographic measurements and corrected the manuscript. AP and SG participated in the echocardiographic measurements. RS and TL carried out the right heart catheterisations. EB, PM and GG participated in the design and the coordination of the study. ES conceived the study, performed the statistical analysis and participated in its design and coordination. All authors have read and approved the final manuscript.
